# Marriage Squeeze, Never-Married Proportion, and Mean Age at First Marriage in China

**DOI:** 10.1007/s11113-013-9283-8

**Published:** 2013-04-30

**Authors:** Quanbao Jiang, Marcus W. Feldman, Shuzhuo Li

**Affiliations:** 1 Institute for Population and Development Studies, School of Public Policy and Administration, Xi’an Jiaotong University, Xi’an, 710049 Shaanxi China; 2 Morrison Institute for Population and Resource Studies, Stanford University, Stanford, CA 94305 USA

**Keywords:** Marriage squeeze, Proportion never-married, Mean age at first marriage, Age-specific first-marriage rate, First-marriage frequency

## Abstract

China’s sex ratio imbalance and the surplus of males have received a great deal of attention, but measures of the extent of the marriage squeeze do not take into account the marital status of population. In this paper, we devise an index of the marriage squeeze for the never-married population and use it to project the male marriage squeeze from 2000 to 2060. From the predicted population and nuptiality tables, we estimate trends in the proportion of men that never marry by age 50 and the mean age at first marriage. We find that the marriage squeeze is much more intense if only the never-married population is considered, rather than including all people without distinguishing their marital status. As the lifelong never-married proportion increases, mean age at first marriage rises first and then declines.

## Introduction

China’s sex ratio imbalance and its surplus of males have received a great deal of attention. The imbalance between marriageable males and females entails that some males or females will be unable to choose their spouse according to the generally accepted criteria, and a number of people will fail to marry at all. Beginning in 2010, annually there will be a large number of young males who cannot find a Chinese spouse, and China will be confronted with a serious male marriage squeeze for decades. More than 10 % of males born after 1980, when China’s stringent fertility policy was implemented, will not be able to find a spouse. The number of surplus males between the ages of 20 and 49 will continue to increase, reaching 20 million by 2015, 30 million by 2025, and 40 million by 2040 (Chen 2004). China’s *National Population Development Strategy Report* estimates that there will be 30 million more males than females for people aged 20–45. Poston et al. (2011) estimated that between 1983 and 2020, China will have produced a total of at least 51 million surplus males.

In China, females traditionally change their status through marriage, usually by choosing a spouse of higher status, i.e., hypergamous marriage. With the increase of female marriage migration, the majority of males who are unable to marry belong to the lowest social strata (Hesketh and Zhu 2006). In the early 1980s, only 0.5 % of males with college education or above were unmarried by the age of 40, but the proportion was 15 % for the illiterate and semiliterate; in 1990, the proportion of poor farmers who did not marry by age 40 was 19 %; in 2000, 44.8 % of males aged 30 who had the least education had never married (Wang and Mason 2006). In the 2000 census, the proportion of never-married males in the age group 40–44 who are illiterate is 29.3 %; while for males of the same age and with primary school education it is 7.4 %, and males with junior middle school education who remain single account for 2.2 %. In the 2010 census, 37.90 % of illiterate males of this same age group, 8.67 % of males of primary school education, and 2.75 % of males with junior middle school education were unmarried (Population Census Office under the State Council 2002, 2012). These males are often popularly referred to as “bare branches”, and a bare-branch class, consisting of 40–50 million males, will come into being at the lowest strata of Chinese society (Chen 2006). In many poverty-stricken remote rural areas of several provinces, the numbers of bare branches have increased and “bare-branch villages” have formed (Jiang and Sánchez-Barricarte 2013). Further increase in their number could affect the nation’s sustainable development.

The effect of surplus males on socio-economic development has been investigated (Hudson and den Boer 2004; Edlund et al. 2007; Ebenstein and Sharygin 2009; Das Gupta et al. 2010; Wei and Zhang 2011), while other studies have focused on the marriage squeeze (Tuljapurkar et al. 1995; Ebenstein and Sharygin 2009; Jiang et al. 2011b). These studies have tended to focus on the sex ratio of a cohort or an age group as a whole, or have calculated a weighted sex ratio. However, such calculations do not take into consideration the marital status of the population. Guilmoto (2012) focused on the effects of China’s marriage squeeze on single individuals and on marriage patterns. In fact, the majority of the population, males or females, can marry, and those who are squeezed in the marriage market are unmarried males at the bottom of the social hierarchy (Hesketh and Zhu 2006). In addition, the marriage squeeze will result in an increase in the lifelong never-married population. As they age, the never-married elderly will place strong demands on social security due to lack of care from wives and children, their poverty, and their comparatively lower social status (Ebenstein and Sharygin 2009; Das Gupta et al. 2010). It is important to estimate the effects of the marriage squeeze on the lifelong never-married proportion in order to project the old-age needs of the social security system. A second issue related to the male marriage squeeze is variation in the mean age at first marriage. The matching process between males and females in the marriage market is in many ways similar to that between employers and employees in the labor market. Just as the lack of employment opportunities extends the time during which the unemployed search for work, the shortage of women available for marriage extends the time it takes for males to find a spouse. Market constraints, such as the sex ratio imbalance, will increase the difficulty of finding a wife and delay the timing of marriage (Oppenheimer 1988).

In this paper, we explore the influence of the marriage squeeze on the proportion of males who will never marry and on the mean age at first marriage for males in China. We devise a marriage squeeze index for the never-married population; we estimate the extent of the male marriage squeeze from 2000 to 2060; we also estimate the lifelong never-married proportion and the mean age at first marriage for males. One contribution of our article is methodological. In much of the scholarly and popular literature on China’s marriage squeeze or excess males, the methodology is not provided (Poston et al. 2011). Our method is different from those in studies that did provide a method in that we use a longitudinal simulation to predict the proportion of never-married males from a cohort perspective. Besides results obtained from population projection and simulation, we also use the nuptiality table to obtain period indicators for future years. In addition, we measure the tightness of the squeeze on never-married males, as they are the group that will be confronted with problems in the marriage market. It turns out that if we restrict the marriage squeeze to never-married males, the extent of the marriage squeeze is more severe than if the total population (i.e., not distinguishing their marital status) is considered. As the lifelong never-married proportion increases, the mean age at first-marriage first rises and then declines. We first devise an index to measure the marriage squeeze for the never-married population, and then introduce the data, parameters, and assumptions. The baseline data used in this paper are from the 2000 census. We then present our results, and conclude with some discussion.

## Method

### Sex Ratio of Potential First-Marriage Partners

One of the commonly used indices to estimate the marriage squeeze is the sex ratio, and many studies restrict the population studied to a small age range (Goldman et al. 1984; Lampard 1993; Ebenstein and Sharygin 2009). One advantage of such indices lies in their easy calculation, but they only estimate the sex ratios of age-specific groups or the number of surplus males for a specific age difference between males and females. In reality, the range of ages at marriage is quite wide. Tuljapurkar et al. (1995) used the sex ratio of potential first-marriage partners to predict the tightness of China’s marriage squeeze. Under the assumption that first-marriage frequencies and patterns for the years subsequent to the baseline year remain unchanged, the index was computed as the ratio of the age-specific number of males, weighted by the age-specific first-marriage frequencies for males, to the age-specific number of females, weighted by the corresponding frequencies for females. The age-sex-specific first-marriage frequencies in a given year are defined as the ratios of the numbers of first marriages of the specific ages and sex during a certain period to the corresponding total population of the same ages and sex. Jiang et al. (2011b) attempted to improve this index by eliminating the tempo effect (the influence of change in event timing, such as advancing or delaying marriage, on statistical indicators (Bongaarts and Feeney 1998, 2002) on first-marriage frequencies and by normalizing the total first-marriage rates for both sexes, because they are well below unity. However, these revised indicators also do not consider marital status. An example will help to illustrate our focus in this paper. If 120 males and 100 females are born, then the general sex ratio is 120 males to 100 females in the marriage market without considering gender differentials in mortality. After 80 couples are married there are 40 males and 20 females left in the marriage market. For these never-married males, the marriage pressure is much more severe than that experienced by the whole population without considering their marital status.

Before introducing our new index, we first distinguish two kinds of age-specific first-marriage rate. The first-marriage rate of the first kind (occurrence/exposure rate) is the age-specific first-marriage rate for never-married persons, calculated as the ratio of the number of first marriages in an age group to the never-married population in the same age group. These age-specific first-marriage rates can be used to construct a nuptiality table from which we can derive the proportions never married up to different ages, and the mean duration of stay in the unmarried state for those who ultimately marry, namely the mean age at first marriage. The second kind of first-marriage rate is the ratio of the number of age-specific first marriages to the total population in that age group, which is also called the first-marriage frequency. As the married population is not exposed to the risk of first marriage, and the competition in the first-marriage market will only be faced by the never-married population, the second kind of first-marriage rate obscures or even distorts the demand and supply relationship in the marriage market. Here, we devise a sex ratio to measure the marriage squeeze for those who never marry.

We denote the number of males and females of age *x* in the year *t* in the whole population by *P*_*t*_^*m*,*x*^ and *P*_*t*_^*f*,*x*^, respectively; the number of never-married males and females by ${\text{NM}}_{t}^{m,x}$ and ${\text{NM}}_{t}^{f,x}$, respectively; and first-marriage rates of the first kind by ${\text{FMR}}_{t}^{m,x}$ and ${\text{FMR}}_{t}^{f,x}$ for males and females, respectively. In demography, a person who is never married by age 50 is regarded as lifelong never married, so we restrict our age range for first-marriage rates from 14 to 49. Taking the year 2000 as the baseline year, the sex ratio of potential first-marriage partners can be expressed intuitively as *R*_*t*_^′^:

1 $$R_{t}^{{^{\prime } }} = \frac{{\sum\nolimits_{x = 14}^{49} {{\text{NM}}_{t}^{m,x} \times {\text{FMR}}_{2000}^{m,x} } }}{{\sum\nolimits_{x = 14}^{49} {{\text{NM}}_{t}^{f,x} \times {\text{FMR}}_{2000}^{f,x} } }}. $$

This index has the following defects. First, it does not take into account the influence of the relative number of males and females in the baseline year. Without considering the two-sex problem, and remarriage, we assume the number of never-married males of each age in the baseline year is twice that of females, and that first-marriage rates of the first kind for males of each age are one-half of those for females. The calculated sex ratio of potential marriage partners in the baseline year is one, indicating that the number of male first marriages in this year equals that of females. However, since there are twice as many marriageable never-married males, males are severely squeezed in the marriage market. Obviously, the sex ratio of one in this example fails to reflect the severity of marriage squeeze. A second flaw lies in the use of age-specific first-marriage rates of the first kind obtained directly from the census data, which may be affected by many other factors. In fact, if we replace the first kind of first-marriage rates by the second kind in the year 2000, we can calculate the total first-marriage rates in 2000, denoted by ${\text{TFMR}}_{2000}^{m}$ and ${\text{TFMR}}_{2000}^{f}$, respectively, namely:

$${\text{TFMR}}_{2000}^{m} = \sum\limits_{x = 14}^{49} {{\text{FMR}}_{2000}^{m,x} } /({\text{NM}}_{2000}^{m,x} /P_{2000}^{m,x} ) = 0.736$$

$${\text{TFMR}}_{2000}^{f} = \sum\limits_{x = 14}^{49} {{\text{FMR}}_{2000}^{f,x} } /({\text{NM}}_{2000}^{f,x} /P_{2000}^{f,x} ) = 0.780.$$

In addition to underreporting of first marriages and increase in the lifelong never-married proportion, which contribute to the low total first-marriage rates for males and females from the 2000 census data, another important contributor may be the tempo effect in marriage. In 1975, the mean age of brides’ first marriages was from 22 to 24 in some Western countries, but in the mid-1990s it was between 26 and 29 years, and the mean age of males’ first marriages was also higher (Kiernan 2001). Older age at marriage can explain the sharp fall in marriage rates in Western countries (Winkler-Dworak and Engelhardt 2004). Coale et al. (1991) noted the effect of changes in marriage ages on period first-marriage rates in China. When the mean age at first-marriage rises, the total first-marriage rate falls in the corresponding period of time, and the opposite occurs if there is a fall in the age at first marriage. Therefore, the observed TFMR, which is affected by delayed or advanced timing of marriage, fails to reflect the lifelong completed total first-marriage rate for a specific cohort. In addition, if ${\text{FMR}}_{2000}^{f,x}$ is used, the estimated lifelong never-married proportion for females is $\prod\limits_{x = 14}^{49} {(1 - {\text{FMR}}_{2000}^{f,x} } ) = 0.012$. In fact, the actual proportions of women never married at ages 45–49 in 2000 in China are 2.24, 2.25, 1.98, 1.90, and 1.85 per thousand, respectively. The estimate 0.012 is five to seven times higher than the actual proportions.

There would be a discrepancy in the results if the age-specific first-marriage rates obtained in the baseline year 2000 applied directly, so it is necessary to adjust those rates. We take $c_{m}$ and $c_{f}$ as normalizing coefficients for males and females, and normalize the total first-marriage rates in the baseline year 2000 as follows:

$$\begin{gathered} {}_{\text{norm}}{\text{TFMR}}_{2000}^{m} = c_{m} \sum\limits_{x = 14}^{49} {{\text{FMR}}_{2000}^{m,x} } /(NM_{2000}^{m,x} /P_{2000}^{m,x} ) = 1, \\ {}_{\text{norm}}{\text{TFMR}}_{2000}^{f} = c_{f} \sum\limits_{x = 14}^{49} {{\text{FMR}}_{2000}^{f,x} } /({\text{NM}}_{2000}^{f,x} /P_{2000}^{f,x} ) = 1 \hfill \\ \end{gathered}$$

The female lifelong never-married proportion is $\prod\limits_{x = 14}^{49} {(1 - c_{f} {\text{FMR}}_{2000}^{f,x} } ) = 0.0033$ from 2000 census data after normalization, which is closer to those for females aged 45–49 in 2000 than the above estimate of 0.012. Then, assuming women’s first-marriage rates do not change from 2000 to 2060, the sex ratio of future potential first-marriage partners is:

2 $$R_{t} = \frac{{\sum\nolimits_{x = 14}^{49} {{\text{NM}}_{t}^{m,x} \times c_{m} \times {\text{FMR}}_{2000}^{m,x} } }}{{\sum\nolimits_{x = 14}^{49} {{\text{NM}}_{t}^{f,x} \times c_{f} \times {\text{FMR}}_{2000}^{f,x} } }} = R_{t}^{'} (c_{m} /c_{f} ). $$

Here *R*_*t*_ is employed to measure the marriage squeeze. We can see that this indicator is different from those in previous studies (Tuljapurkar et al. 1995; Jiang et al. 2011a, b) in that it takes into account only the never-married population when calculating the tightness of the marriage squeeze as it is the never-married who face the pressure in the marriage market. In order to trace the never-married population, we use a longitudinal simulation method, distinguishing the population in the baseline year by age, sex, and marital status (two categories, namely never-married and others), and then project them to the next year using demographic parameters.

In reality the number of male marriages should equal that of female marriages, namely,

$$\frac{{\sum\nolimits_{x = 14}^{49} {{\text{NM}}_{t}^{m,x} \times {\text{FMR}}_{t}^{m,x} } }}{{\sum\nolimits_{x = 14}^{49} {{\text{NM}}_{t}^{f,x} \times c_{f} \times {\text{FMR}}_{2000}^{f,x} } }} = 1$$

where *c*_*f*_ is computed as above from 2000 census data. Assuming the age-specific first-marriage rates for males are scaled by the same factor, we can estimate the age-specific first-marriage rates in a specific future year as ${\text{FMR}}_{t}^{m,x} = {{c_{m} \times {\text{FMR}}_{2000}^{m,x} } \mathord{\left/ {\vphantom {{c_{m} \times {\text{FMR}}_{2000}^{m,x} } {R_{t} }}} \right. \kern-0pt} {R_{t} }}$. Then we can obtain ${\text{NM}}_{t + 1}^{m,x + 1}$ as

3 $${\text{NM}}_{t + 1}^{m,x + 1} = {\text{NM}}_{t}^{m,x} \times (1 - {\text{FMR}}_{t}^{m,x} ) \times {{L_{x + 1} } \mathord{\left/ {\vphantom {{L_{x + 1} } {L_{x} }}} \right. \kern-0pt} {L_{x} }} , $$

where *L*_*x*_ is the person years aged from *x* to *x* + 1 in the life table. We apply the relationship between rate and probability (Keyfitz and Caswell 2005) to obtain the probability *q*_*t*_^*m*,*x*^ that a male’s first marriage occurs at time *t* when his age is *x*: $q_{t}^{m,x} = {{2 \times {\text{FMR}}_{t}^{m,x} } \mathord{\left/ {\vphantom {{2 \times {\text{FMR}}_{t}^{m,x} } {(2 + {\text{FMR}}_{t}^{m,x} )}}} \right. \kern-0pt} {(2 + {\text{FMR}}_{t}^{m,x} )}}$.

We can find the probability of first marriage, and then construct annual nuptiality tables for males.

### Lifelong Never-Married Proportion for Males

We use two methods to estimate the proportion of lifelong never-married at each time *t*. One is based on the population projection: from formula (3) we can calculate the age-specific never-married proportion in future years, and take $\left[ {{{{\text{NM}}_{t}^{m,50} } \mathord{\left/ {\vphantom {{{\text{NM}}_{t}^{m,50} } {P_{t}^{m,50} }}} \right. \kern-0pt} {P_{t}^{m,50} }}} \right] \times 100$ at age 50 as the lifelong never-married percentage. This result applies for every birth cohort when they reach age 50; thus it can be regarded as a cohort indicator. The other way is to obtain the proportion through the generated male nuptiality tables: $l_{50} = \prod\limits_{x = 14}^{49} {(1 - q_{t}^{m,x} } )$ in each year, where *l*_50_ is the proportion of men who never married by age 50 in a nuptiality table, and *q*_*t*_^*m*,*x*^ is the first-marriage probability of men aged *x* at time *t*. As probabilities for all ages in the nuptiality table are obtained for that year, the proportion never-married can be regarded as a period indicator.

### Mean Age at First Marriage for Males

Two methods are used to calculate the mean age at first marriage for males. One is through the predicted population, from which we can obtain the never-married proportion by age, and derive the singulate mean age at marriage (SMAM). The sum of all the proportions of never-married men from ages 14 to 49 is

$${\text{RS}}_{1} = \left( {\sum\limits_{x = 14}^{49} {{\text{NM}}_{t}^{m,x} /P_{t}^{m,x} } } \right)$$

Define

$${\text{RS}}_{2} = {\text{RS}}_{1} + 14.0,$$

and denote the never-marrying proportion before the age of 50 as

$${\text{RM}} = 1.0 - {\text{NM}}_{t}^{m,50} /P_{t}^{m,50}.$$

Then the duration of singlehood for those never-married at age 50 is

$${\text{RS}}_{3} = 50.0 \; \times \;{\text{NM}}_{t}^{m,50} /P_{t}^{m,50},$$

and the singulate mean age at marriage is

$${\text{SMAM}} = \left( {{\text{RS}}_{2} - {\text{RS}}_{3} } \right)/{\text{RM}} .$$

Another method is to use 14 + *e*_14_, which is based on the expected duration of singlehood *e*_14_ at age 14 in the male nuptiality table.

## Data

### Population Size and Structure in the Baseline Year

The release of the 2010 population census data led to a debate about data quality, and doubt about the reliability of China’s population data since 1990. Population data, estimation, and projection from 1990 to 2010 generally exaggerate the number of births; they overestimate fertility level and population growth (Guo 2011). In *World Population Prospects*—*the 2010 Revision*, China’s population size for 2010 is adjusted downward by 13 million, from 1,354 million in *the 2008 Revision* to 1,341 million in *the 2010 Revision*. China’s population size and fertility rate have been overestimated during the past two decades (Cai 2012), and the 2010 census data themselves are not perfect. For example, the sex ratio of males to females for people aged 20–24 is 98.95, and that for the 25–29 age group is 98.84, a significant sign of misreporting that is probably due to migration.

In this paper, the baseline year adopted is 2000, and we use birth data published in the years subsequent to 2000. We fit the total population with different parameters to insure, for example, that total fertility rates are made as close as possible to reality. The 2000 census was basically successful, with an underreporting rate of just 1.81 %. Although by international standards, this underreporting rate is reasonable (Walfish 2001), the birth numbers for each year released before the 2000 census do not agree with the 2000 census data, especially in the young age groups. For example, the released numbers of births were 20.38, 19.1, and 19.09 million, respectively, in the sample surveys of 1997, 1998, and 1999, respectively. However, the population at the age of 0, 1, and 2 was 13.79, 11.50, and 14.01 million, respectively, in the 2000 census. Even the sample survey data themselves are not consistent. For example, from 2001 to 2009 the released annual number of births is about 16 million, but it was more than 19 million before 2000. We adjusted the numbers in the younger groups from the 2000 census, to make the birth numbers projected from the baseline data fit the released birth numbers taken from the sample surveys in years 2001 to 2009 (Jiang et al. 2011a). The number of never-married in each age group in 2000 can be derived using the proportion of never-married obtained from the long form multiplied by the population number in each age group.

### Total Fertility Rate and Sex Ratio at Birth

In the 1990s and early 2000s, scholars were concerned that the numbers of annual births released by China National Bureau of Statistics were underestimated (Merli and Raftery 2000; Attané 2001). However, further research has reached the consensus that the annual numbers of births were actually overestimated (Zeng 2007; Cai 2008; Morgan et al. 2009; Goodkind 2011). The total fertility rate was 1.22 in the 2000 census, while Morgan et al. (2009) estimate the total fertility in 2000 to have been between 1.4 and 1.6, which is similar to estimates from many other studies (Zhang and Zhao 2006; Gu et al. 2007; Cai 2008). In *World Population Prospects*—*The 2010 Revision*, the total fertility rate for China drops from 1.7 for 2000–2005 to 1.64 for 2005–2010, and then it declines further to 1.51 for 2015–2020. Recently, major population projection units such as the Population Reference Bureau and the US Census Bureau International Programs have adjusted China’s total fertility rate since 2000 to around 1.5 (Cai 2012). Furthermore, even if China’s family planning policy were suspended, the total fertility rate would not change much (Cai et al. 2010; Merli and Morgan 2010). If we set the TFR at 1.5 from 2000, the forecast births are about 1 million less than the 16 million reported by the National Bureau of Statistics for the first several years after 2000. Hence we fix the TFR at 1.6 in 2000, and reduce it by 0.01 annually. After 2010, we take a constant TFR of 1.50, with 0.99 for the first birth, 0.50 for the second birth, and 0.01 for the third birth (Jiang et al. 2011a). This makes the projected population size 0.36 % less than the released census result for 2010, which is acceptable.

The sex ratio at birth plays a key role in determining the future squeeze in the marriage market. China’s sex ratios at birth have fluctuated around 120 since 2000. Using household registration data from the Ministry of Public Security, and school enrollment data from Ministry of Education, Zhai and Yang (2011) claim that actual SRBs may be 2–3 % point lower than that reported. Based on data from the census and intercensal samples, some researchers have observed an incipient decline in the sex ratio at birth in China, as in South Korea (Das Gupta et al. 2009; Guilmoto 2009). According to the National Population and Family Planning Commission, China’s SRB has declined for four consecutive years, from 120.56 in 2008, to 119.45 in 2009, 117.94 in 2010, 117.78 in 2011, and 117.70 in 2012 (Li 2013). However, the sex ratio at birth from preliminary results of the 2010 census stands at 118.6, higher than the more reliable estimate of 116.9 from the 2000 census short form, while that from the 2000 long form is 119.9, a statistic more often cited but less reliable (Goodkind 2011). Whether the sex ratio at birth will decline, or remain at around 120 is currently uncertain. We take SRB_*j*_, the sex ratio at birth of the *j*th child, in the year 2000 to be: SRB_1_ = 107.1, SRB_2_ = 151.9, SRB_3+_ = 159.4. We assume that the sex ratios at birth for different parities from 2000 to 2010 remain at the level of 2000, and fall linearly from 2010 to 2040 until they become normal, i.e., 106, in 2040 and after.

### Age-Specific First-Marriage Rates

**Fig. 1:**
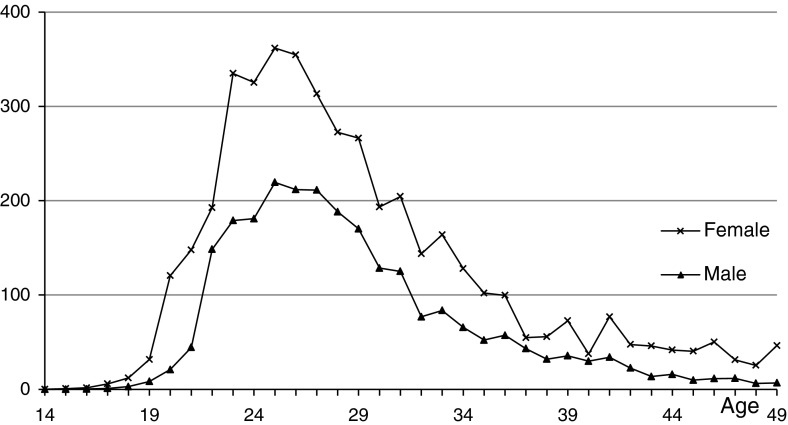
Age-specific first-marriage rates in 2000 (per thousand)

Marriage data from the census of 2000 are available. The age-specific first-marriage rates are shown in Fig. 1.

We use the traditional cohort-component method to predict age-sex-specific numbers. Based on the calculated numbers of first marriages by age, we retrieve annual numbers of the never-married population by age and sex.

## Results

### The Sex Ratio of Potential First-Marriage Partners

**Fig. 2:**
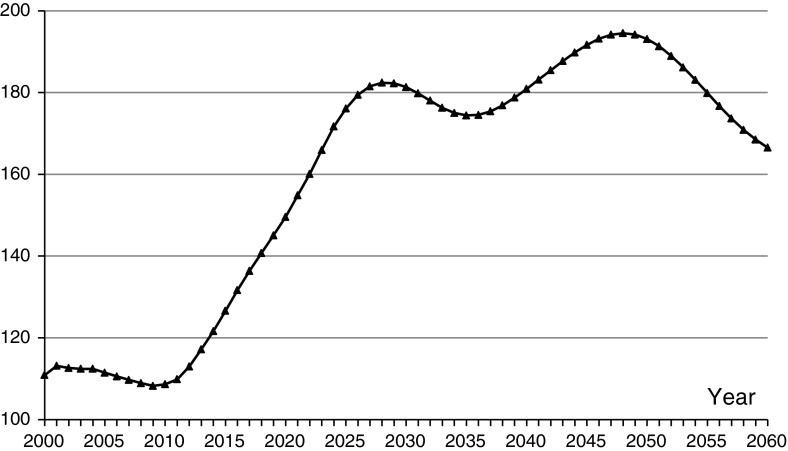
Sex ratio

Choice of a spouse will be affected by social, economic, cultural, ethnic, and individual conditions. But the basic property of a population is the numbers of males and females. Because China’s fertility rates have fallen rapidly since the 1980s, leading to a decline in the size of subsequent birth cohorts, the marriage market is not only determined by the sex ratio imbalance but also by the changing age structure (Goodkind 2006; Jiang et al. 2011b). On the other hand, the sex ratio imbalance can, in the long run, have a significant influence on the age structure (Attané 2006; Jiang et al. 2011a). We do not decompose the influences of age structure and gender structure on the marriage squeeze in this paper. From Fig. 2, we can see that the sex ratio of potential first-marriage partners begins to grow rapidly after 2010. China’s sex ratio at birth has deviated from normal since 1980, and people born after the 1980s have entered the marriage market gradually, which has caused the sex ratio of first-marriage partners to rise. In 2020, the sex ratio will be 150 never-married males for 100 never-married females, so three never-married males will compete for two never-married females, and this ratio will rise to 180 males for 100 females in 2030; although there are expected to be some fluctuations from 2030 to 2050, it remains high. The sex ratio is close to 200 males for 100 females between 2045 and 2050, and then declines.

### Lifelong Never-Married Proportion

**Fig. 3:**
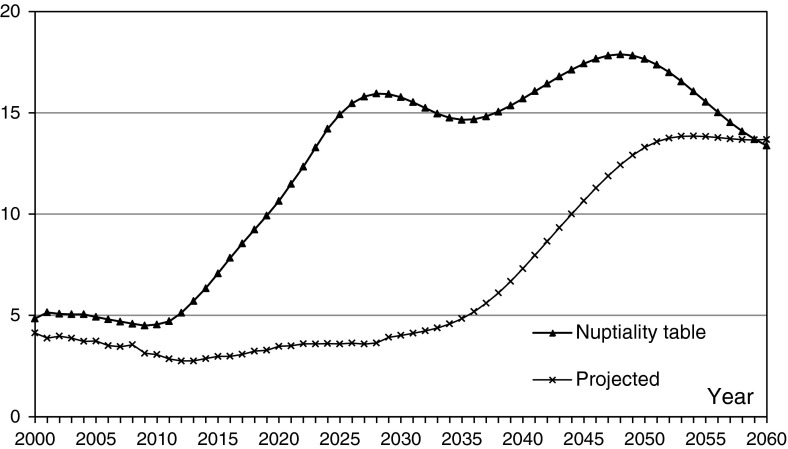
Lifelong never-married percent (%)

As Fig. 3 shows, the lifelong never-married proportions derived from the projected population remain under 5 % before 2035. The proportion between 2000 and 2035 corresponds to the cohort born from 1950 to 1985, whose sex ratio at birth is in the normal range. Near the founding of People’s Republic of China, excess female child mortality due to infanticide was not significant, so the lifelong never-married proportions for the cohorts from that period are normal, under 5 %. However, as the cohorts born after 1985 reach the age of 50, namely, from the year 2035, the lifelong never-married proportion will begin to rise, and will exceed 10 % in 2044 and 13 % in 2050. Because of the assumption that the sex ratio at birth remains unchanged from 2000 to 2010, the lifelong never-married proportion is stable in the period from 2050 to 2060.

On the other hand, from the nuptiality table, the lifelong never-married proportion remains around 5 % from 2000 to 2010, and begins to increase from 2010, exceeding 10 % in 2020, over 15 % in 2026, and then it fluctuates around 15 %. One of the reasons for the difference between the results obtained by population projection and the nuptiality table is that the predicted lifelong never-married proportions are for those cohorts born 50 years earlier, whose marriages occurred 20 or 30 years earlier, so the age-specific first-marriage rates are comparatively high. As explained above, the nuptiality tables are obtained through data that can be regarded as period data, so that the age-specific first-marriage rates are lower than those of 20 or 30 years ago and produce higher lifelong never-married proportions.

### Mean Age at First Marriage

Although changes in the age at first marriage may be caused by wider structural transformations such as changes in level of education and more job opportunities, change in local marriage markets is also important (Xu, Hudspeth, and Bartkowski 2005).In the north of Jiangsu Province during the Qing dynasty, poor men had to delay marriage by 6 years relative to men from well-off families (Ownby 1996). In the late 1920s and early 1930s, an investigation of 143 farmers’ families in Qingyuan County of Shanxi Province showed that women’s average age at first marriage was 16 years, and men’s was 26.2. There were fewer women in Shanxi Province, and men’s marriage had to be delayed (Qiao 1947). If China’s male age at marriage increases and the gap in age between spouses reaches 8 years by 2050, the share of men who fail to ever marry can be kept close to the historical rate of 5 %, even for a 15 % excess of men over women (Ebenstein and Sharygin 2009). Guilmoto (2012) demonstrates the rise in male marriage age with a demographic simulation of multiple marriage models and scenarios in China’s male-squeezed marriage market.

**Fig. 4:**
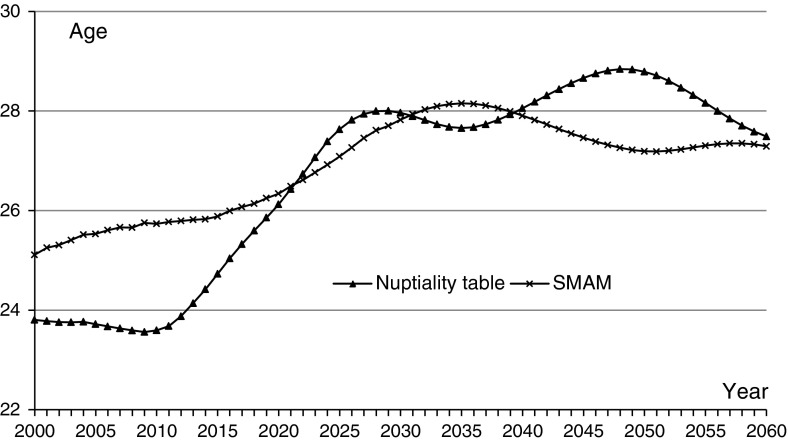
Mean age at first marriage

As can be seen from Fig. 4, the singulate mean age at marriage derived from the population projection rises gradually from 2000, and falls slowly after 2035. In calculating SMAM, the never-married proportion is a very important factor. With the rise in the proportion of lifelong never-married after 2000, SMAM begins to rise, but after this proportion exceeds a certain value, it begins to fall slowly. The mean age at first marriage (actually singlehood duration for those who eventually get married) derived from the nuptiality tables begins to rise rapidly after 2000, is nearly equal to the SMAM in 2020, and falls after 2050.

## Conclusion and Discussion

The strict family planning policy has been in existence for 30 years, and China’s sex ratio at birth is much higher than normal, currently at about 120. The Chinese government has been aware of the severity of the sex ratio imbalance and has taken a series of measures, such as the nationwide “Care for Girls” campaign. There is also a fierce debate in academic circles over relaxing the strict one-child policy. The above indices could play a role in predicting the future sex ratio at birth, but they cannot mitigate the male surplus in China.

We found that the sex ratio for the never-married population in the first-marriage market will exceed 150 males for 100 females after the year 2020. The male surplus could produce various strategies for finding a spouse, and many men have gone to younger female cohorts to seek a spouse. Though the range of spouses can be expanded temporarily, the marriage squeeze for subsequent male cohorts will be more serious because of the rise in the sex ratio at birth and the reduction in numbers of births. Some non-mainstream marriages, such as exchange marriages, mercenary marriages, and child brides, have recently tended to increase. Exchange marriage avoids embarrassment for the two partners who cannot get married; child brides have appeared once again in some poor and backward rural areas; trafficked women and mercenary marriage is a strategy adopted by men at the bottom of society to compete under the marriage squeeze (Chen 2006). The sex ratio imbalance causes more people to remain unmarried, which has an impact on family stability. For example, in Taiwan, the divorce rate was 1.8 % for males aged 40 or above in 1935, and rose to 4.1 % in 1966. There may be ideological explanations for this, but the unbalanced gender structure caused by a high proportion of males in the large migration from mainland China to Taiwan probably played a major role. At present, with the exacerbation of the sex ratio imbalance, divorced women find spouses more easily than divorced men. Hence, the divorce rate in rural areas is increasing and the proportion of women who get a divorce is rising (Shi 2002).

The marriage squeeze will result in more lifelong never-married males. Annually, after 2044 more than 10 % of males aged 50 will never have been married. The majority of rural elderly people rely on their family members for daily care and old age support. With the development of China’s economy, it is hoped that the Chinese government will have the financial power to provide the social security that is presently the family’s responsibility. In 2009, the State Council issued *The Guidance for Developing Pilots of a Social Old*-*Age Support System in Rural Areas*, which promotes rapid development of social protection for the elderly population in rural areas. However, the current coverage and payment capacity are quite low, and cannot meet the needs of the rural elderly population. Even in the near future, the proposed system will not be financially able to cover all the people in need, so the family support system will remain dominant for quite a long time. As more never-married males enter old age, how they will be supported in their old age is an urgent problem faced by the government and society. Never-married elderly males do not have their own family, and the majority of them are not well-off, so they need financial as well as instrumental and emotional support. They will become destitute both in material and in spirit. Bare-branch villages, now scattered around China and increasing rapidly, will soon become “Five Guarantees” villages,^1^ and result in even more pressure on social security and a potential breakdown in social stability (Shi 2006).

People born in the mid-to-late 1980s and after are gradually entering the marriage market, but a certain fraction of them will fail to find a spouse, and will have to postpone marriage; the mean age at first marriage will increase. In addition to social and economic development, delay of marriage because of difficulty in finding a spouse as a result of sex ratio imbalance is a very important factor. Shortage of females increases the bargaining power for females in the marriage market. When females are in short supply, males have to increase their bride price to compete for a spouse (Becker 1991), and families have to provide better houses to enhance the relative attractiveness of a son (Wei and Zhang 2011). The current bride price and marriage expenses are as high as dozens of times the annual income for parents who depend on agricultural production, and is such a burden that many young people migrate to work in cities after junior middle school, to accumulate money for their marriage expenses. But they are disadvantaged in both educational attainment and skills, have little chance to be well paid, and will have difficulty in obtaining enough money to pay for marriage. But by the time they do accumulate some money, they will have become older than the generally accepted marriage age, and become bare branches, which means they will pay more than the usual amount for bride price and expenses for marriage. This vicious cycle, “unable to pay bride price-bare branches-pay more bride price” will entail that many men will fail to ever marry.

When females are in short supply, it is the poor males who suffer from the shortage of spouses (Das Gupta and Li 1999). If a large number of bare branches congregate in poverty-stricken regions, there may be a number of negative consequences. Currently, the bare-branch villages have caught the public’s attention and that of academics. If single males cluster in destitute and backward rural areas, then, triggered by certain events, they may take collective measures to improve their own situation, sharpen social conflicts, and become a potential threat to social stability. This demands that the Chinese government take precautions and preventative countermeasures.

## Footnotes

1 Five Guaranteed people refers to rural elderly people who are unable to work, who have neither sources of income nor family support, or whose family members do not have the ability to support them. It is stipulated that in rural areas, local economic organizations should provide adequate food and clothing, housing and medical service, and proper funeral arrangements (five aspects in all) for these elderly people.
